# Supplementary material for: Mice carrying a GluN2B protein-truncating variant have altered NMDA receptor subunit composition and their behavior recapitulates patient phenotypes
Source: Cell Mol Life Sci. 2026 Jan 30;83(1):89. doi: 10.1007/s00018-025-06057-1 (PMC12876548; doi:10.1007/s00018-025-06057-1)
Supplement: Supplementary file 1 — Supplementary Material 1(DOCX 989 KB) [file 18_2025_6057_MOESM1_ESM.docx]

# Supplementary information

**Supplementary Tables:**

**Table S1 |** Sequences of primers used for PCR analysis and inventory numbers of TaqMan Gene Expresssion Assays used for qPCR.

**Table S2 |** The list of significantly altered proteins and annotation of their functional role.

**Table S3 |** Protein-set enrichment analysis overview (≥2-fold change or ≤−2-fold change).

**Table S4 |** Protein-set enrichment analysis overview (≥1.5-fold change or ≤−1.5-fold change).

**Table S5 |** The list of selected synaptic proteins and their abundance ratio assessed in *Grin2b*^+/Δ^ relative to *Grin2b*^+/+^ hippocampal tissue.

**Table S6 |** Data reported in Fig. 8–10, grouped by sex.

**Table S7 |** Numerical data underlying Fig. 11–12 and Fig. S5–S8.

**Supplementary Figures:**

**Figure S1 |** A representative agarose gel showing amplification of a 1200 nt fragment of *Grin2b* mRNA.

**Figure S2 |** Correlation between protein expression changes in hippocampal tissue from 4-month-old *Grin2b*^+/Δ^ and *Grin2b*^+/L825V^ mice.

**Figure S3 |** **Western blot analysis of HEK293T cells transfected with GFP or GluN2B-GFP subunit.**

**Figure S4 |** The GluN2B^Δ^ subunit disrupts NMDAR surface expression in COS-7 cells.

**Figure S5 |** *Grin2b*^+/Δ^ **mice display hypoactivity and increased anxiety. Supplementary data from the open field test.**

**Figure S6 | Elevated plus maze analysis of anxiety-like behavior in** *Grin2b*^+/Δ^ **mice.**

**Figure S7 | Social behavior in** *Grin2b*^+/Δ^ **mice.**

**Figure S8 | Repetitive behavior in** *Grin2b*^+/Δ^ **mice.**

### Supplementary Tables

| **Gene** | **Primer Forward** | **Primer Reverse** | **Notes** |
| --- | --- | --- | --- |
| **Genotyping PCR** | | | |
| *Grin2b* | GAGGCAAATGGGGAAAAGCC | GGAAGCTCTCTGGCTCACTG |  |
|  | | | |
| **End-point PCR** | | | |
| *Grin2b* | CCTATGACCTTTACCTGGTGACC | GCTGATGGAGAAGACCATGC |  |
| *Grin2b* | TGCAGCCGTGCTCAACTACA |  | Sequencing primer (forward) |
|  | | | |
| **RT-qPCR (TaqMan Gene Expression Assay)** | | | |
| *Grin1* | Mn004338000_m1 | | Thermo Fisher |
| *Grin2a* | Mn004338002_m1 | | Thermo Fisher |
| *Grin2b* | Mn004338020_m1 | | Thermo Fisher |
| *Gapdh* | Mn999999915_m1 | | Thermo Fisher |
| *β2M* | Mm00437762_m1 | | Thermo Fisher |

**Table S1 | Sequences of primers used for PCR analyses and inventory numbers of TaqMan Gene Expresssion Assays used for qPCR.**

| **Protein** | **Protein symbol** | **Annotation of the Functional Role** | **Ratio**  **log_2_ (2b^+/Δ^/2b^+/+^)** |
| --- | --- | --- | --- |
| MDR3 P-glycoprotein | ABCB4 | ABCB4 transports phospholipids across liver cell membranes for release into bile. | 1.0319 |
| Actin-like protein 6B | ACTL6B | Mutations in this protein result in neurodevelopmental deficits, epilepsy, and dendritic loss in human neurons. | 1.1841 |
| Armadillo-like helical domain-containing protein 3 | ARMH3 | Regulates Golgi organization; localized to the Golgi membrane and cytosol. | 1.7692 |
| Beta-site amyloid precursor protein cleaving enzyme 1 | BACE1 | Plays a key role in the production of amyloid-beta peptides implicated in Alzheimer’s disease. | 1.1617 |
| BCL2-associated X protein | BAX | Bax, a pro-apoptotic Bcl-2 family member, triggers apoptosis and is essential for developmental neuronal cell death. | 1.0368 |
| Cell division cycle 37-like 1 protein | CDC37L1 | This protein has been associated with gastric cancer. | 1.2357 |
| Cysteine-serine-rich nuclear protein 1 | CSRNP1 | CSRNP1 is a tumor suppressor, and its expression is increased by estradiol. | 1.1717 |
| Cytochrome P450 family 4 subfamily V member 2 | CYP4V2 | This protein is a cytochrome P450 enzyme involved in oxidizing substrates in metabolism, particularly in converting fatty acid precursors to n-3 polyunsaturated fatty acids. | 1.0131 |
| DEAD-box helicase 3, Y-linked | DDX3Y | It is a crucial RNA helicase involved in RNA processing, translation regulation, and spermatogenesis. | 1.0381 |
| Fibrous sheath-interacting protein 2 | FSIP2 | FSIP2 is involved in acrosome development during spermiogenesis and may serve as a predictive biomarker for Clear Cell Renal Cell Carcinoma prognosis. | 1.085 |
| Growth arrest-specific protein 8 | GAS8 | A component of the cytoskeleton involved in ciliary function and microtubule organization. | -1.212 |
| Histone H2B ubiquitinated 2 | H2BU2 | This histone is involved in chromatin organization and gene regulation. | -1.332 |
| Histone acetyltransferase 1 | HAT1 | Histone acetyltransferase 1 acts as a succinyltransferase for both histones and non-histones, promoting tumorigenesis. | 1.0848 |
| Inositol 1,4,5-trisphosphate receptor type 3 | ITPR3 | It is a calcium channel that mediates intracellular calcium release from the endoplasmic reticulum. | 1.0904 |
| Minichromosome maintenance complex component 4 | MCM4 | The expression of certain MCM complex components, including MCM4, a helicase essential for DNA replication and genome stability, is linked to the survival of hepatocellular carcinoma (HCC) patients. | 1.1244 |
| Metallothionein 1 | MT1 | This protein is involved in heavy metal detoxification, oxidative stress response, and metal ion homeostasis. | -1.3131 |
| Myosin light chain kinase | MYLK | Encodes the Ca2+/calmodulin-dependent myosin light chain kinase (MLCK), responsible for phosphorylating the regulatory light chain and initiating smooth muscle cell contraction. | 1.0677 |
| Myoferlin | MYOF | Myoferlin is involved in membrane repair and fusion processes in muscle cells and is overexpressed in various cancers, particularly pancreatic and triple-negative breast cancer. | 1.1297 |
| N-deacetylase/N-sulfotransferase 4 | NDST4 | This protein is a candidate tumor suppressor involved in heparan sulfate modification, which regulates cellular signaling and development. | 1.0822 |
| Optic atrophy 3 protein | OPA3 | This protein plays a role in mitochondrial function and is associated with optic atrophy. | 1.0101 |
| Ribosomal RNA Processing Protein 1 | RRP1 | This is a nucleolar protein that helps in processing and assembling ribosomal RNA into functional ribosomes. | -1.0416 |
| Translocation protein SEC62 | SEC62 | This protein is involved in the translocation of nascent proteins across the endoplasmic reticulum membrane. | 1.5698 |
| Vacuole membrane protein 1 | VMP1 | It is involved in autophagy and vacuole membrane formation and is overexpressed in pancreatitis-affected acinar cells, where it mediates zymophagy. | 1.5755 |

**Table S2 | The list of significantly altered proteins and the annotation of their functional role.**

| **Term** | **Library** | **p-value** | **q-value (FDR)** | **Protein** |
| --- | --- | --- | --- | --- |
| Positive regulation of neuron apoptotic process (GO:0043525) | *a* | 0.0003438 | 0.06230 | BACE1; BAX |
| Positive regulation of neuron death (GO:1901216) | *a* | 0.001325 | 0.06230 | BACE1; BAX |
| Histone H4 acetylation (GO:0043967) | *a* | 0.001382 | 0.06230 | ACTL6B; HAT1 |
| Histone acetylation (GO:0016573) | *a* | 0.002996 | 0.06230 | ACTL6B; HAT1 |
| Regulation of neuron apoptotic process (GO:0043523) | *a* | 0.005623 | 0.06230 | BACE1; BAX |
| Increased hepatocellular carcinoma incidence MP:0003331 | *b* | 0.001943 | 0.07983 | ABCB4; MCM4 |
| Postnatal lethality, incomplete penetrance MP:0011086 | *b* | 0.004172 | 0.07983 | BACE1; ACTLB; MCM4; GAS8 |
| Abnormal locomotor activation MP:0003313 | *b* | 0.004262 | 0.07983 | BACE1; ITPR3 |
| Abnormal myelination MP:0000920 | *b* | 0.00436 | 0.07983 | BACE1; ACTL6B |
| Decreased fibroblast proliferation MP:0011704 | *b* | 0.00446 | 0.07983 | HAT1; MCM4 |
| Release of apoptotic factors from mitochondria R-HSA-111457 | *c* | 0.008023 | 0.1722 | BAX |
| CLEC7A (Dectin-1) induces NFAT activation R-HSA-5607763 | *c* | 0.01258 | 0.1722 | ITPR3 |
| Unwinding of DNA R-HSA-176974 | *c* | 0.01258 | 0.1722 | MCM4 |
| Elevation of cytosolic Ca2+ Levels R-HSA-139853 | *c* | 0.01712 | 0.1722 | ITPR3 |
| Signaling by NTRK3 (TRKC) R-HSA-9034015 | *c* | 0.01825 | 0.1722 | BAX |
| Gastric acid secretion | *d* | 0.003424 | 0.1780 | ITPR3; MYLK |
| Platelet activation | *d* | 0.00886 | 0.1780 | ITPR3; MYLK |
| Vascular smooth muscle contraction | *d* | 0.01013 | 0.1780 | ITPR3; MYLK |
| Apelin signaling pathway | *d* | 0.01073 | 0.1780 | ITPR3; MYLK |
| Apoptosis | *d* | 0.01149 | 0.1780 | BAX; ITPR3 |

**Table S3 |** **Protein-set enrichment analysis overview (≥2-fold change or ≤−2-fold change).** The table presents the results of enrichment analysis for proteins showing a ≥2-fold increase or ≤−2-fold decrease across two ontologies, *a* – GO_Biological_Process_2021 and *b* – MGI_Mammalian_Phenotype_Level_4_2021, and two pathway databases, *c* – Reactome_2022 and *d* – KEGG_2021_Human. The analyses were performed using Enrichr (<https://maayanlab.cloud/enrichr-kg>). Enriched terms were ranked according to their FDR to assess the significance of biological processes and pathways associated with the analyzed protein set. Columns list, in order: term name (protein set) with reference identifier, the corresponding ontology or pathway database, *p*-value (Fisher’s exact test), and *q*-value/FDR (Benjamini–Hochberg correction).

| **Term** | **Library** | **p-value** | **q-value (FDR)** | **Protein** |
| --- | --- | --- | --- | --- |
| Negative regulation of microtubule polymerization (GO:0031115) | a | 0.0000936 | 0.06084 | CADH5; STMN1; DYRK1A |
| Negative regulation of microtubule polymerization or depolymerization (GO:0031111) | a | 0.0001028 | 0.06084 | CADH5; STMN1; DYRK1A; HDGFL3 |
| Cellular component assembly (GO:0022607) | a | 0.0001954 | 0.07712 | VMP1; TTC26; ARL6IP1; FLNA; RHOH; TUBGCP4; PARVB; GAS8; LIMS1 |
| Abnormal blood homeostasis MP:0009642 | b | 0.0001993 | 0.2151 | CFH; ARG1; ABCB4; OPA3; RDX; ALPL |
| Cell-extracellular matrix interactions R-HSA-446353 | c | 0.0002329 | 0.1043 | FLNA; PARVB; LIMS1 |
| Increased circulating aspartate transaminase level MP:0005343 | b | 0.0005943 | 0.2498 | PON3; ATXN3; ABCB4; RDX; FACE1 |
| Regulation of protein modification process (GO:0031399) | a | 0.0006376 | 0.1509 | CDK9; CADH5; FINC; PUM3 |
| Focal adhesion | d | 0.0009542 | 0.1584 | LAMA1; COL6A1; FINC; FLNA; FLNB; PARVB; MYLK |
| Incomplete embryo turning MP:0001701 | b | 0.001038 | 0.2498 | CADH5; GLMN; FINC; MGAT1 |
| Negative regulation of protein polymerization (GO:0032272) | a | 0.001143 | 0.1509 | CADH5; STMN1; DYR1A |
| Abnormal thrombosis MP:0005048 | b | 0.001143 | 0.2498 | CFAH; FLNA; PDLI7 |
| Abnormal artery development MP:0003410 | b | 0.0016 | 0.2498 | PKHG5; FLNB |
| Regulation of cytoskeletal remodeling and cell spreading by IPP complex components R-HSA-446388 | c | 0.0016 | 0.3584 | PARVB; LIMS1 |
| Cell junction organization R-HSA-446728 | c | 0.004402 | 0.5327 | CADH5; FLNA; PARVB; LIMS1 |
| Signaling by receptor tyrosine kinases R-HSA-9006934 | c | 0.005151 | 0.5327 | CADH5; LAMA1; COL6A1; STMN1; BAX; ITPR3; RICTR; T2FB; GLUN2B; JAK3 |
| Proteoglycans in cancer | d | 0.005165 | 0.4287 | RADI; FINC; FLNA; MOES; FLNB; ITPR3 |
| Sensory processing of sound by outer hair cells of cochlea R-HSA-9662361 | c | 0.007915 | 0.5327 | CLIC5; RADI; MOES |
| Spinocerebellar ataxia | d | 0.02478 | 0.6944 | ATX3; ITPR3; ATG13; GLUN2B |
| ECM-receptor interaction | d | 0.03054 | 0.6944 | LAMA1; COL6A1; FINC |
| Small-cell lung cancer | d | 0.03419 | 0.6944 | LAMA1; FINC; BAX |

**Table S4 |** **Protein-set enrichment analysis overview (≥1.5-fold change or ≤−1.5-fold change).** The table presents the results of enrichment analysis for proteins showing a ≥1.5-fold increase or ≤−1.5-fold decrease across two ontologies, *a –* GO_Biological_Process_2021 and *b* – MGI_Mammalian_Phenotype_Level_4_2021, and two pathway databases, *c* – Reactome_2022 and *d* – KEGG_2021_Human. The analyses were performed using Enrichr (<https://maayanlab.cloud/enrichr-kg>). Enriched terms were ranked according to their FDR to assess the significance of biological processes and pathways associated with the analyzed protein set. Columns list, in order: term name (protein set) with reference identifier, the corresponding ontology or pathway database, *p*-value (Fisher’s exact test), and *q*-value/FDR (Benjamini–Hochberg correction).

| **Protein name** | **Protein symbol** | **Ratio**  **(*Grin2b*^+/Δ^ /*Grin2b*^+/+^)** |  |
| --- | --- | --- | --- |
| Disks large homolog 2 | DLG2 | 1.03 |  |
| Disks large homolog 3 | DLG3 | 1.01 |  |
| Disks large homolog 4 | DLG4 | 1.12 |  |
| Disks large-associated protein 1 | DLGAP1 | 1.10 |  |
| Disks large-associated protein 2 | DLGAP2 | 1.03 |  |
| Disks large-associated protein 3 | DLGAP3 | 0.98 |  |
| Disks large-associated protein 4 | DLGAP4 | 1.00 |  |
| Homer protein homolog 1 | HOMER1 | 1.08 |  |
| Homer protein homolog 2 | HOMER2 | 1.09 |  |
| Homer protein homolog 3 | HOMER3 | 1.26 |  |
| Neurexin-1 | NRXN1 | 1.04 |  |
| Neurexin-2 | NRXN2 | 1.08 |  |
| Neurexin-3 | NRXN3 | 1.11 |  |
| Neuroligin-2 | NLGN2 | 1.03 |  |
| Neuroligin-3 | NLGN3 | 1.14 |  |
| Protein bassoon | BSN | 0.90 |  |
| Protein piccolo | PCLO | 0.95 |  |
| Regulating synaptic membrane exocytosis protein 1 | RIMS1 | 1.09 |  |
| Regulating synaptic membrane exocytosis protein 2 | RIMS2 | 1.00 |  |
| SH3 and multiple ankyrin repeat domains protein 1 | SHANK1 | 1.17 |  |
| SH3 and multiple ankyrin repeat domains protein 2 | SHANK2 | 1.10 |  |
| SH3 and multiple ankyrin repeat domains protein 3 | SHANK3 | 1.16 |  |
| Synapsin-1 | SYN1 | 1.08 |  |
| Synapsin-2 | SYN2 | 1.23 |  |
| Synapsin-3 | SYN3 | 0.83 |  |
| Synaptic vesicle glycoprotein 2A | SV2A | 0.90 |  |
| Synaptic vesicle glycoprotein 2B | SV2B | 1.12 |  |
| Synaptic vesicle membrane protein VAT-1 | VAT-1 | 1.31 |  |
| Synaptic vesicle membrane protein VAT-1homolog-like | VAT-1I | 0.90 |  |
| Synaptobrevin homolog YKT6 | YKT6 | 0.97 |  |
| Synaptophysin | SYP | 1.09 |  |
| Synaptophysin-like protein 1 | SYPL1 | 1.27 |  |
| Synaptosomal-associated protein 25 | SNAP25 | 0.95 |  |
| Synaptosomal-associated protein 47 | SNAP47 | 1.10 |  |
| Synaptotagmin-1 | SYT1 | 1.14 |  |
| Synaptotagmin-11 | SYT11 | 1.21 |  |
| Synaptotagmin-12 | SYT12 | 1.13 |  |
| Synaptotagmin-17 | SYT17 | 1.04 |  |
| Synaptotagmin-2 | SYT2 | 0.92 |  |
| Synaptotagmin-3 | SYT3 | 1.04 |  |
| Synaptotagmin-7 | SYT7 | 1.14 |  |
| Syntaxin-12 | STX12 | 1.07 |  |
| Syntaxin-16 | STX16 | 1.38 |  |
| Syntaxin-17 | STX17 | 1.05 |  |
| Syntaxin-1A | STX1A | 1.06 |  |
| Syntaxin-1B | STX1B | 1.04 |  |
| Syntaxin-4 | STX4 | 1.16 |  |
| Syntaxin-5 | STX5 | 1.15 |  |
| Syntaxin-8 | STX8 | 1.22 |  |
| Syntaxin-binding protein 1 | STXBP1 | 0.89 |  |
| Syntaxin-binding protein 3 | STXBP3 | 1.12 |  |
| Syntaxin-binding protein 5 | STXBP5 | 1.05 |  |
| Syntaxin-binding protein 5-like | STXBP5L | 1.11 |  |
| Vesicle-associated membrane protein 1 | VAMP1 | 1.10 |  |
| Vesicle-associated membrane protein 2 | VAMP2 | 1.18 |  |
| Vesicle-associated membrane protein 4 | VAMP4 | 1.53 |  |
| Vesicle-associated membrane protein 7 | VAMP7 | 1.19 |  |
| Vesicle-associated membrane protein-associated protein A | VAPA | 1.10 |  |
| Vesicle-associated membrane protein-associated protein B | VABP | 0.99 |  |
| Vesicular glutamate transporter 1 | VGLUT1 | 1.07 |  |
| Vesicular glutamate transporter 2 | VGLUT2 | 0.72 |  |

**Table S5 | The list of selected synaptic proteins and their abundance ratio in *Grin2b*^+/Δ^ relative to *Grin2b*^+/+^ hippocampal tissue.**

|  | ***Grin2b*^+/+^** | | | ***Grin2b*^+/Δ^** | | |
| --- | --- | --- | --- | --- | --- | --- |
|  | **Male** | **Female** |  | **Male** | **Female** |  |
|  | **Mean ± SEM (*n*)** | **Mean ± SEM (*n*)** | ***p*-value** | **Mean ± SEM (*n*)** | **Mean ± SEM (*n*)** | ***p*-value** |
| **Agonist induced whole-cell currents** | | | | | | |
| NMDA current density (pA/pF) | 52.0 ± 3.4 (32) | 48.3 ± 5.3 (22) | 0.17 | 29.1 ± 3.7 (17) | 26.6 ± 2.2 (42) | 0.49 |
| AMPA current density (pA/pF) | 43.3 ± 4.5 (27) | 54.4 ± 13.2 (13) | 0.93 | 35.0 ± 4.6 (14) | 33.4 ± 2.4 (43) | 0.64 |
| Ifenprodil inhibition (%) | 24.8 ± 2.5 (32) | 25.3 ± 3.8 (19) | 0.91 | 18.5 ± 2.8 (17) | 17.7 ± 1.9 (41) | 0.72 |
| **EPSCs** | | | | | | |
| NMDA eEPSC current density (pA/pF) | 17.2 ± 3.1 (35) | 21.5 ± 6.9 (21) | 0.85 | 20.5 ± 4.5 (32) | 12.6 ± 3.0 (19) | 0.75 |
| AMPA eEPSC current density (pA/pF) | 36.9 ± 5.0 (36) | 37.3 ± 8.5 (22) | 0.8 | 39.8 ± 6.3(33) | 34.7 ± 7.5 (21) | 0.90 |
| Ifenprodil inhibition (%) | 31.1 ± 2.5 (25) | 33.0 ± 2.9 (19) | 0.71 | 31.3 ± 3.0 (25) | 29.5 ± 5.7 (15) | 0.77 |
| Tau weighted (ms) | 208 ± 11 (36) | 238 ± 16 (21) | 0.12 | 147 ± 8 (32) | 142 ± 9 (18) | 0.69 |
| Paired-pulse ratio | 0.91 ± 0.05 (28) | 1.00 ± 0.06 (20) | 0.24 | 1.03 ± 0.06 (26) | 0.92 ± 0.04 (17) | 0.28 |
| **Spontaneous activity** | | | | | | |
| Frequency (Hz) | 3.7 ± 1.0 (21) | 3.1 ± 0.9 (17) | 0.7 | 4.2 ± 1.1 (28) | 2.8 ± 1.2 (19) | 0.04 |
| Amplitude (pA) | 18.6 ± 2.1 (21) | 20.3 ± 1.7 (17) | 0.27 | 28.8 ± 2.0 (28) | 24.6 ± 2.4 (19) | 0.07 |

**Table S6 | Data reported in Fig. 8-10, grouped by sex.** Data from 13–43 neurons per group (*Grin2b*^+/+^ and *Grin2b*^+/Δ^) are presented as mean ± SEM. Reported *p*-values refer to the comparisons between sexes within genotypes (Student’s t-test).

| **Test** | | **Male** | | | **Female** | | |
| --- | --- | --- | --- | --- | --- | --- | --- |
|  |  | ***Grin2b*^+/+^** | ***Grin2b*^+/Δ^** |  | ***Grin2b*^+/+^** | ***Grin2b*^+/Δ^** |  |
|  |  | **Mean ± SEM (*n*)** | **Mean ± SEM (*n*)** | ***p*-value** | **Mean ± SEM (*n*)** | **Mean ± SEM (*n*)** | ***p*-value** |
| **Open field test** | Whole arena average speed (m/s) | 3.8 ± 0.2 (10) | 2.9 ± 0.2 (11) | 0.044 | 3.9 ± 0.2 (12) | 4.0 ± 0.2 (10) | 0.89 |
|  | Periphery distance travelled (m) | 165.8 ± 1.0 (10) | 13.3 ± 0.9 (11) | 0.19 | 16.8 ± 0.9 (12) | 17.9 ± 1.0 (10) | 0.54 |
|  | Periphery resting time (s) | 419 ± 12 (10) | 467 ± 13 (11) | 0.02 | 430 ± 11 (12) | 451 ± 12 (10) | 0.44 |
|  | Periphery permanence time (s) | 502 ± 12 (10) | 548 ± 12 (11) | 0.039 | 519 ± 11 (12) | 541 ± 12 (10) | 0.24 |
|  | Periphery average speed (m/s) | 3.2 ± 0.2 (10) | 2.7 ± 0.2 (11) | 0.15 | 3.3 ± 0.2 (12) | 3.4 ± 0.2 (10) | 0.79 |
|  | Center resting time (s) | 58 ± 8 (10) | 35 ± 5 (11) | 0.08 | 46 ± 6 (12) | 35 ± 5 (10) | 0.29 |
|  | Center permanence time (s) | 95 ± 11 (10) | 56 ± 6 (11) | 0.03 | 82 ± 8 (12) | 64 ± 7 (10) | 0.22 |
|  | Whole arena resting time (s) | 482 ± 6 (10) | 507 ± 5 (11) | 0.03 | 477 ± 5 (12) | 483 ± 6 (10) | 0.56 |
|  | Center distance travelled (m) | 7.0 ± 0.5 (10) | 4.5 ± 0.5 (11) | 0.001 | 7.0 ± 0.5 (12) | 6.2 ± 0.5 (10) | 0.43 |
|  | Number of center entries | 46.6 ± 3.1 (10) | 34.0 ± 2.9 (11) | 0.016 | 45.7 ± 2.8 (12) | 44.8 ± 3.1 (10) | 0.89 |
|  | Total distance travelled (m) | 22.8 ± 1.2 (10) | 17.8 ± 1.2 (11) | 0.04 | 23.8 ± 1.1 (12) | 24.2 ± 1.2 (10) | 0.86 |
|  | Center average speed (m/s) | 7.5 ± 0.5 (10) | 6.3 ± 0.5 (11) | 0.23 | 8.4 ± 0.5 (12) | 9.5 ± 0.5 (10) | 0.22 |
|  | Latency to center entry (s) | 16.8 ± 3.9 (10) | 22.9 ± 5.1 (11) | 0.43 | 12.5 ± 2.7 (10) | 33.8 ± 7.9 (10) | 0.04 |
| **IntelliCage system** | Activity | - | - | - | 51.6 ± 1.5 (9) | 51.8 ± 1.5 (8) | 0.93 |
|  | Place preference learning (%) | - | - | - | 41.3 ± 1.1 (9) | 40.3 ± 1.1 (8) | 0.44 |
|  | Place preference reversal (%) | - | - | - | 73.9 ± 0.9 (9) | 69.3 ± 0.9 (8) | 0.26 |
|  | Place preference extinction (%) | - | - | - | 27.2 ± 1.3 (9) | 24.6 ± 1.2 (8) | 0.11 |
|  | Patrolling (%) | - | - | - | 26.9 ± 0.6 (9) | 27.8 ± 0.6 (8) | 0.31 |
| **Freezing time** | Baseline (%) | 1.1 ± 0.4 (10) | 1.9 ± 0.8 (11) | 0.64 | 1.2 ± 0.5 (12) | 0.4 ± 0.3 (10) | 0.18 |
|  | Context (%) | 0.8 ± 0.2 (10) | 0.9 ± 0.2 (11) | 0.89 | 1.5 ± 0.3 (12) | 1.8 ± 0.4 (10) | 0.59 |
|  | Cue (%) | 7.5 ± 2.4 (10) | 10.8 ± 2.5 (11) | 0.34 | 15.7 ± 6.0 (12) | 12.7 ± 2.9 (10) | 0.49 |
| **PPI** | 70 dB (%) | 46.6 ± 1.9 (11) | 29.6 ± 2.4 (11) | <0.001 | 46.2 ± 1.9 (12) | 35.9 ± 2.2 (8) | < 0.001 |
|  | 78 dB (%) | 56.6 ± 1.8 (11) | 51.4 ± 1.8 (11) | >0.05 | 55.8 ± 1.5 (12) | 48.1 ± 1.6 (11) | < 0.001 |
|  | 82 dB (%) | 66 ± 2 (11) | 53 ± 2 (11) | <0.001 | 56 ± 2 (12) | 56 ± 2 (11) | >0.05 |
|  | 85 dB | 63 ± 2 (11) | 58 ± 2 (11) | >0.05 | 58 ± 2 (12) | 65 ± 1 (11) | < 0.001 |
| **Elevated plus maze (time** **spent in each zone)** | Open arms (s) | 18 ± 1 (15) | 26 ± 2 (11) | 0.09 | 31 ± 4 (16) | 35 ± 5 (12) | 0.51 |
|  | Closed arms (s) | 238 ± 11 (15) | 235 ± 12 (11) | 0.09 | 222 ± 21 (16) | 200 ± 22 (12) | 0.51 |
|  | Center zone (s) | 34.6 ± 1.9 (15) | 39.9 ± 2.7 (11) | 0.09 | 27.9 ± 3.3 (16) | 37.1 ± 4.9 (12) | 0.51 |
| **Elevated plus maze (visits)** | Open arms | 6.2 ± 0.6 (15) | 5.3 ± 0.7 (11) | 0.17 | 5.0 ± 0.5 (16) | 8.2 ± 0.8 (12) | 0.019 |
|  | Closed arms | 12.9 ± 0.6 (15) | 11.4 ± 0.7 (11) | 0.17 | 11.6 ± 0.8 (16) | 13.1 ± 0.9 (12) | >0.05 |
| **Elevated plus maze (distance)** | Open arms (m) | 1.6 ± 0.3 (15) | 1.6 ± 0.4 (11) | 0.45 | 1.9 ± 0.3 (16) | 1.7 ± 0.3 (12) | 0.98 |
|  | Closed arms (m) | 12.2 ± 0.3 (15) | 11.5 ± 0.4 (11) | 0.45 | 11.4 ± 0.7 (16) | 12.1 ± 0.8 (12) | 0.98 |
| **Object recognition** | Duration familiar (s) | 6.7 ± 1.7 (15) | 3.6 ± 1.3 (11) | 0.28 | 18.3 ± 3.4 (16) | 10.2 ± 2.5 (12) | 0.15 |
|  | Duration novel (s) | 4.7 ± 0.9 (15) | 5.5 ± 1.2 (11) | 0.74 | 5.7 ± 0.9 (16) | 4.9 ± 1.1 (12) | 0.71 |
|  | Discrimination index | 0.60 ± 0.04 (15) | 0.6 ±0.1 (11) | 0.32 | 0.80 ± 0.03 (16) | 0.60 ± 0.04 (12) | 0.02 |
| **Social discrimination** | Duration juvenile (s) | 73.4 ± 7.8 (16) | 64.3 ± 9.4 (11) | 0.59 | 63.6 ± 7.8 (16) | 64.3 ± 9.4 (11) | 0.59 |
|  | Duration object (s) | 28.8 ± 4.2 (16) | 18.8 ± 4.2 (11) | 0.15 | 37.8 ± 4.7 (16) | 21.5 ± 4.5 (11) | 0.14 |
|  | Discrimination index | 0.70 ± 0.03 (16) | 0.80 ± 0.03 (11) | 0.22 | 0.60 ± 0.03 (16) | 0.80 ± 0.03 (11) | 0.01 |
| **Nestlet shredding** | % of shredded nestlet | 18.4 ± 2.2 (15) | 14.7 ± 2.2 (11) | 0.42 | 10.9 ± 1.5 (16) | 7.6 ± 1.4 (12) | 0.21 |

**Table S7 | Numerical data underlying Fig. 11–12 and Fig. S5–S8.** Data from 8–16 animals per group (*Grin2b*^+/+^ and *Grin2b*^+/Δ^ mice) are presented as mean ± SEM. Genotype comparisons within each sex (male and female) were made using ANOVA. Reported *p*-values indicate statistical differences between genotypes within each sex.Supplementary Figures


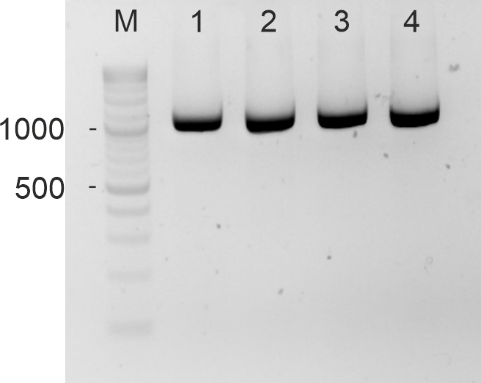


**Figure S1 | A representative agarose gel showing amplification of a 1200 nt fragment of *Grin2b* mRNA.** The mRNA was obtained from hippocampal tissue of *Grin2b*^+/Δ^ female mice at 4 months of age. The forward primer was located in exon 7 and the reverse primer in exon 13 (see Table S1 for sequences). For direct Sanger sequencing of the PCR amplicons, an additional forward primer located in exon 12 was used (see Table S1). M denotes the molecular weight marker; lanes 1–4 represent samples from four individual mice.


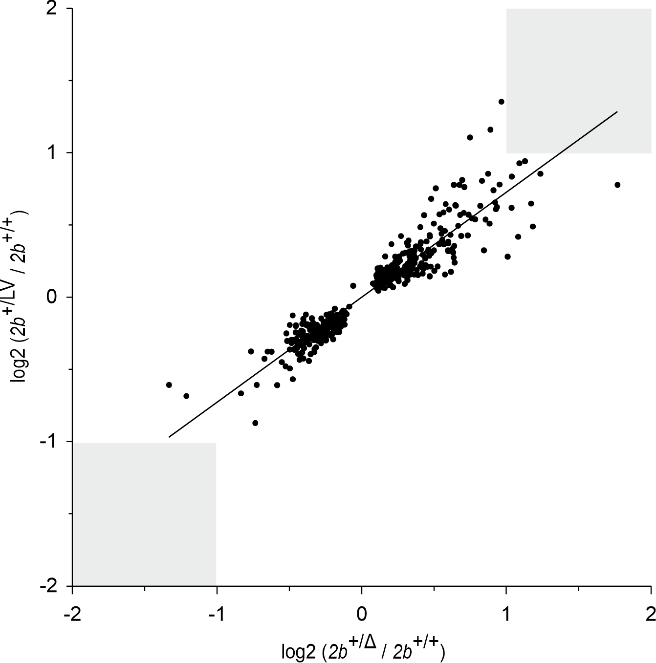


**Figure S2 | Correlation between protein expression changes in hippocampal tissue from 4-month-old *Grin2b*^+/Δ^ and *Grin2b*^+/L825V^ mice.** The scatter plot shows log₂-fold changes for *Grin2b*⁺^/Δ^ / *Grin2b*⁺^/^⁺ and *Grin2b*⁺^/L825V^ / *Grin2b*⁺^/^⁺ mice (Pearson *r* = 0.939, *p* < 0.001; *n* = 461). No proteins exhibited a ≥2-fold increase or decrease in both genotypes, as indicated by the grey rectangles.


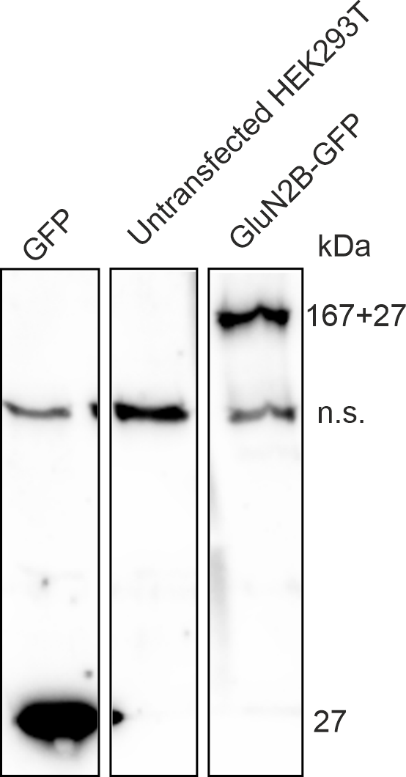


**Figure S3 |** **Western blot analysis of HEK293T cells transfected with GFP or GluN2B-GFP subunit. Note the non-specific band at ~120 kDa (n.s.).**


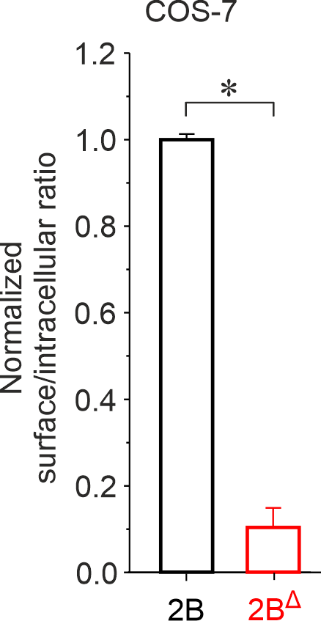


**Figure S4 | GluN2B^Δ^ subunit disrupts NMDAR surface expression in COS-7 cells.** COS˗7 cells were co-transfected with GluN1 and wild-type GluN2B-GFP (2B) or mutant GluN2B^Δ^-GFP (2B^Δ^) subunits. The graph shows the relative surface expression of GluN1/GFP-GluN2B and GluN1/GluN2B^Δ^-GFP receptors, quantified using a colorimetric assay. Data are presented as mean ± SEM. Asterisk (*) indicates a statistically significant difference (Student's t-test; *n* = 4 independent experiments, with the relative surface expression measured in quadruplicate per experiment).


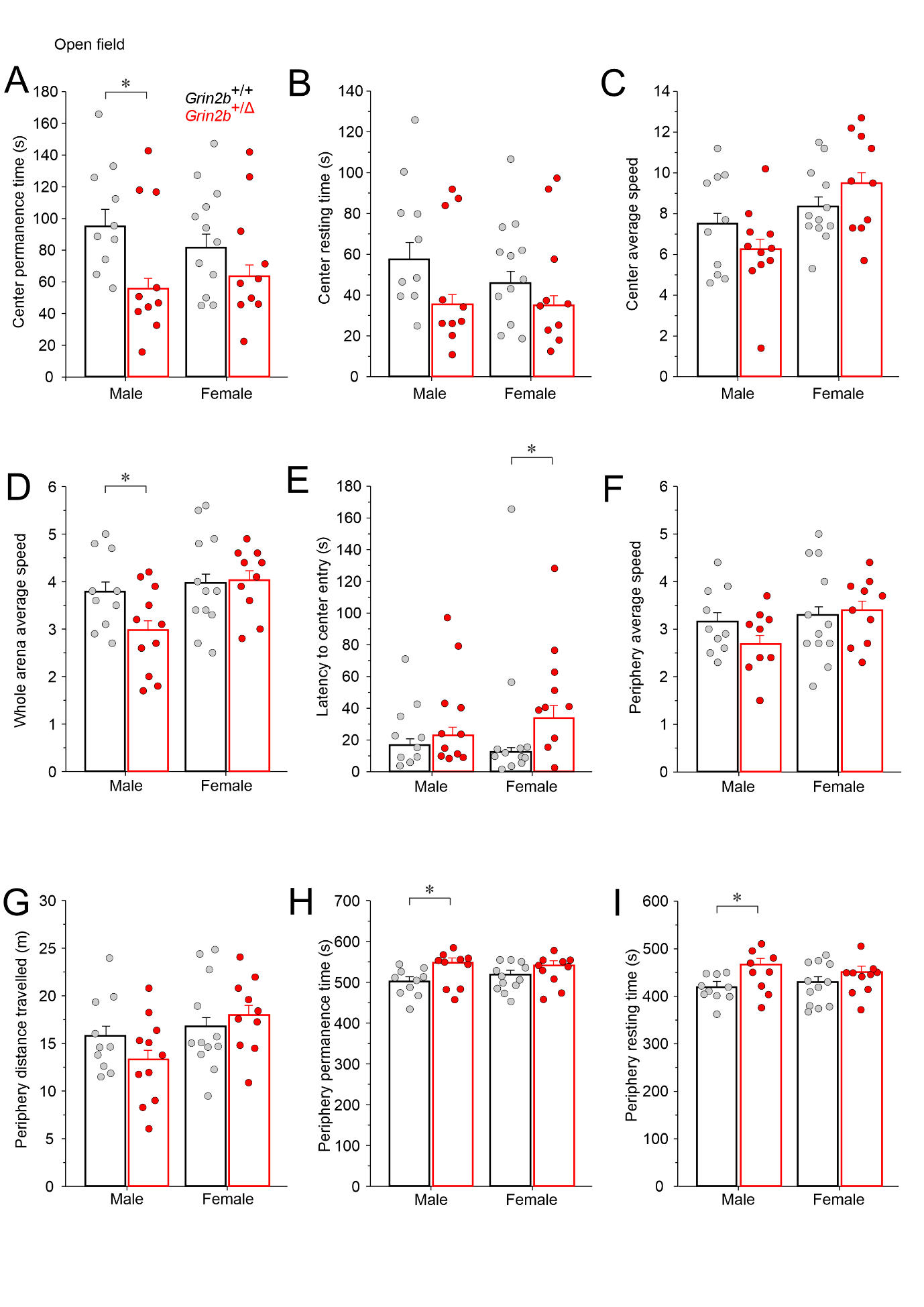


**Figure S5 | *Grin2b*^+/Δ^ mice display hypoactivity and increased anxiety. Supplementary data from the open field test.** (A–I) Center permanence time, center resting time, center average speed, whole arena average speed, latency to first center entry, periphery average speed, periphery distance traveled, periphery permanence time, and periphery resting time were measured in male and female *Grin2b*^+/Δ^ and *Grin2b*^+/+^ mice. Data are presented as mean ± SEM. Number of animals *Grin2b*^+/+^/*Grin2b*^+/Δ^: *n* = 10/11 males and *n* = 12/10 females. Asterisks (*) indicate significant genotype differences (Student’s t-test). The numerical data underlying this figure are shown in Table S7.


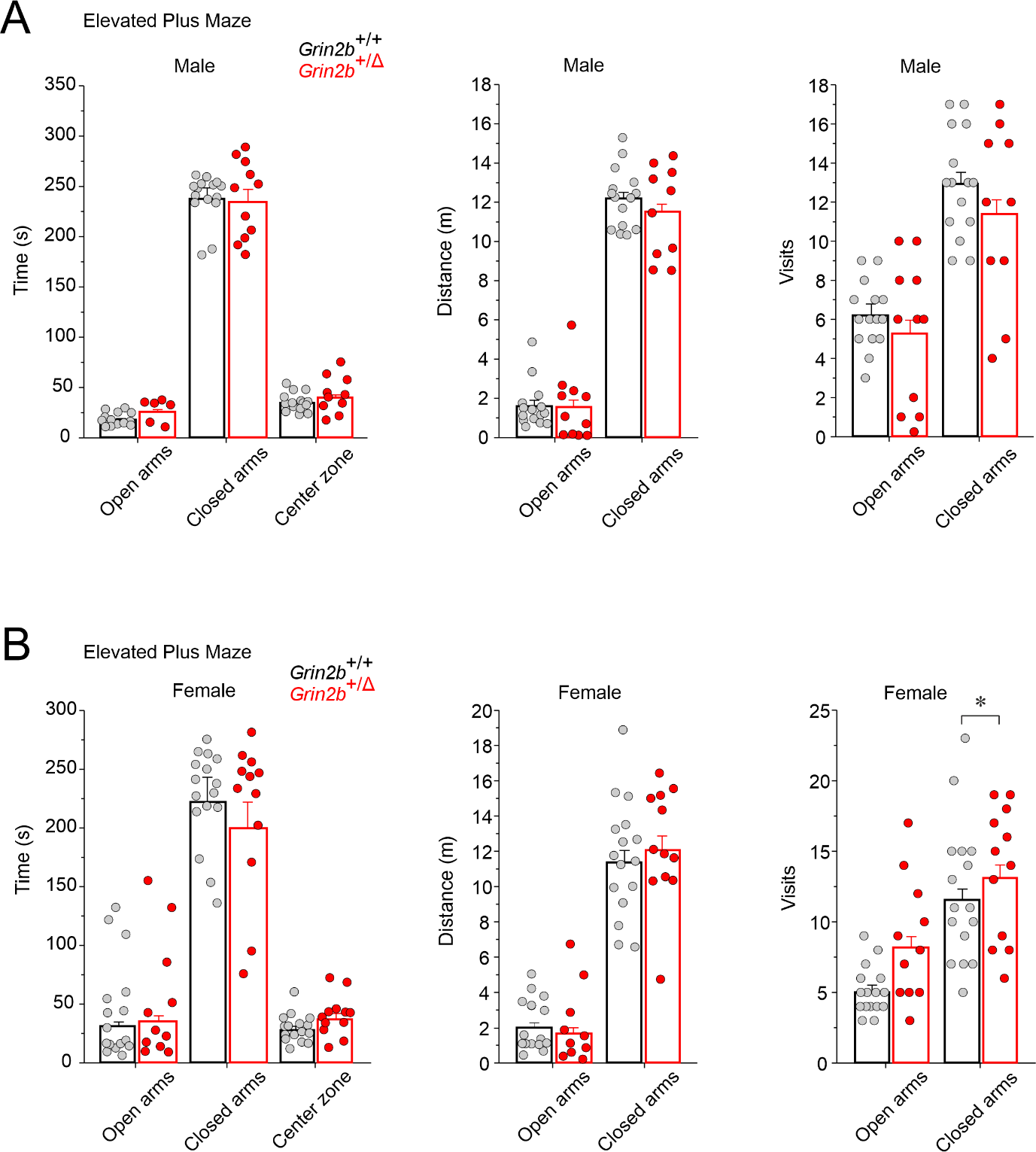


**Figure S6 | Elevated plus maze analysis of anxiety-like behavior in *Grin2b*^+/Δ^ mice.** Bar graphs show measures of anxiety for male (A) and female (B) *Grin2b*^+/Δ^ mice, including time spent, distance traveled, and number of entries into the closed and open arms. Data are presented as mean ± SEM and were analyzed by ANOVA followed by post hoc LSD test. Number of animals *Grin2b*^+/+^/*Grin2b*^+/Δ^: *n* = 15/11 males and *n* = 16/12 females. Asterisk (*) indicates a significant genotype difference. The numerical data underlying this figure are shown in Table S7.


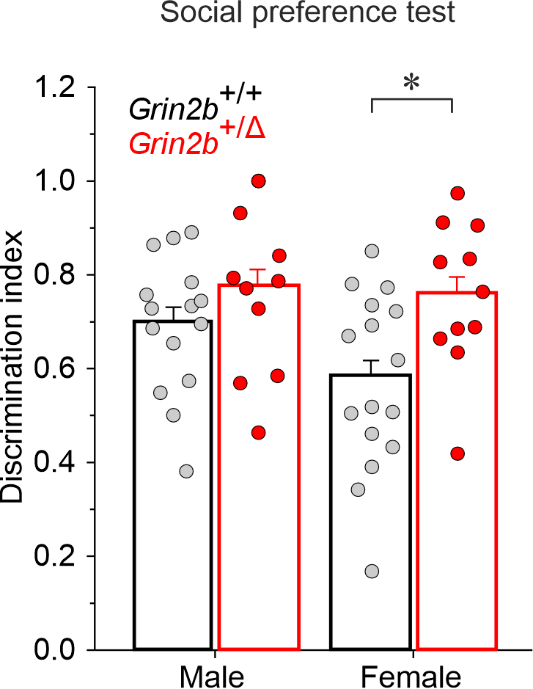


**Figure S7 | Social behavior in *Grin2b*^+/Δ^ mice.** Social preference was assessed, with the discrimination index representing the time spent exploring a juvenile target relative to an object. Data are presented as mean ± SEM and were analyzed by Student’s t-test. Number of animals *Grin2b*^+/+^/*Grin2b*^+/Δ^: 16/11 males, 16/11 females. Asterisk (*) indicates a significant genotype difference. The numerical data underlying this figure are shown in Table S7.


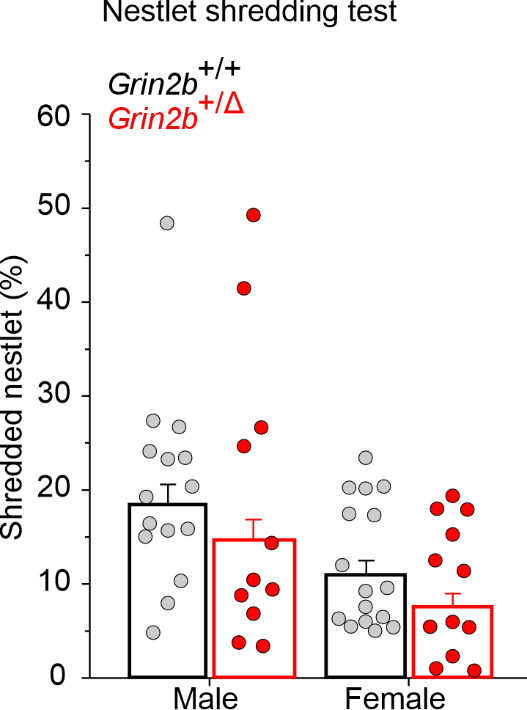


**Figure S8 | Repetitive behavior in *Grin2b*^+/Δ^ mice.** Performance in the nestlet shredding test, measured as the % weight of unused nesting material (initial weight = 10 g). Data are presented as mean ± SEM and were analyzed by Student's *t-test*. Number of animals *Grin2b*^+/+^/*Grin2b*^+/Δ^: *n* = 15/11 males, *n* = 16/12 females. The numerical data underlying this figure are provided in Table S7.
